# Missing Race and Ethnicity Data among COVID-19 Cases in Massachusetts

**DOI:** 10.1007/s40615-022-01387-3

**Published:** 2022-09-02

**Authors:** Keith R. Spangler, Jonathan I. Levy, M. Patricia Fabian, Beth M. Haley, Fei Carnes, Prasad Patil, Koen Tieskens, R. Monina Klevens, Elizabeth A. Erdman, T. Scott Troppy, Jessica H. Leibler, Kevin J. Lane

**Affiliations:** 1 Department of Environmental Health, Boston University School of Public Health, Boston, MA USA; 2 Department of Biostatistics, Boston University School of Public Health, Boston, MA USA; 3 MA Department of Public Health, Bureau of Infectious Disease and Laboratory Sciences, Boston, MA USA; 4 MA Department of Public Health, Office of Population Health, Boston, MA USA

**Keywords:** COVID-19, Health equity, Data collection, Race and ethnicity, Surveillance epidemiology

## Abstract

Infectious disease surveillance frequently lacks complete information on race and ethnicity, making it difficult to identify health inequities. Greater awareness of this issue has occurred due to the COVID-19 pandemic, during which inequities in cases, hospitalizations, and deaths were reported but with evidence of substantial missing demographic details. Although the problem of missing race and ethnicity data in COVID-19 cases has been well documented, neither its spatiotemporal variation nor its particular drivers have been characterized. Using individual-level data on confirmed COVID-19 cases in Massachusetts from March 2020 to February 2021, we show how missing race and ethnicity data: (1) varied over time, appearing to increase sharply during two different periods of rapid case growth; (2) differed substantially between towns, indicating a nonrandom distribution; and (3) was associated significantly with several individual- and town-level characteristics in a mixed-effects regression model, suggesting a combination of personal and infrastructural drivers of missing data that persisted despite state and federal data-collection mandates. We discuss how a variety of factors may contribute to persistent missing data but could potentially be mitigated in future contexts.

## Introduction

Infectious disease surveillance epidemiology in the USA has long had deficiencies in the collection of race and ethnicity data [1], complicating efforts to identify health disparities and enact antiracist interventions to reduce inequities. Over the last two decades, both private and public entities have made attempts to garner more complete demographic data on disease burdens and healthcare use, including collection by health insurers [2], federal reporting to the Department of Health and Human Services [3, 4], and a patchwork of state regulations on data collection [5].

Despite these changes, surveillance systems used to track coronavirus disease 2019 (COVID-19) surveillance systems still showed 42% of nationwide cases with unknown or missing race and ethnicity as of March 2021 [6]. These incomplete demographic data are problematic because there are clear indications of disproportionate impacts of COVID-19 infections, hospitalizations, and deaths among Black, Latinx, and Indigenous communities [7–10], substantially caused by longstanding health inequities from differential access to healthcare, exposure to environmental hazards, and other consequences of systemic racism [11–13]. Moreover, these stark racial and ethnic disparities may in fact be underestimates because there is mounting evidence that these data are not missing at random [14]. While others have documented the issue of missing race and ethnicity data in COVID-19 cases overall [15–19], no analysis to date has analyzed “missingness” as an outcome or identified its individual- and population-scale determinants. Consequently, there is a need to characterize these drivers as an important first step in describing the full extent of COVID-19 health disparities.

In this analysis, we aimed to (1) determine whether missing race and ethnicity data for COVID-19 cases changed over time and varied among towns during the first year of the pandemic in Massachusetts (MA); (2) identify the statistically significant individual- and town-level factors, if any, associated with these missing data; and (3) assess the extent to which these factors changed between the two “waves” of cases that occurred between March 2020 and February 2021. In the analysis that follows, we provide insight about drivers of missingness at multiple spatial scales, discuss challenges that impede race/ethnicity data collection, and offer suggestions for how to improve data collection to advance health equity in infectious disease surveillance epidemiology.

## Methods

### Conceptual Framework

Missing data in COVID-19 cases can arise for a variety of reasons at multiple scales, including preferences at the individual level, provider actions in healthcare settings, and policies at the jurisdictional/town level. Missingness can occur at multiple points in the data-collection process, which generally involved the following steps during our study period: (1) an individual filled out paperwork prior to being tested, which may or may not ask for demographic data; (2) a sample was collected by a healthcare provider or testing site, who may or may not ask for demographic data; (3) the sample was sent to a lab, tested, and entered into a database that may have different reporting options for demographic data than what was collected by the healthcare provider; (4) results were transferred to the Massachusetts Virtual Epidemiologic Network (MAVEN) at the Department of Public Health [20]; and (5) information on positive cases was sent to the local board of health (LBOH) in the patient’s city or town of residence, which then either conducted its own case investigation or contact tracing (CI/CT) or referred the cases to contractors in the Community Tracing Collaborative [21].

We built a multilevel regression model using covariates that were best equipped to capture the variability in the individual and structural sources of missingness in this process. First, we recognized that some amount of missingness will be due to an individual’s choice not to provide demographic information, in which case the data will be missing regardless of any institutional or infrastructural circumstances. We included all individual-level characteristics available in the data set (described in the “Individual-Level COVID-19 Case Data” section) to capture as much person-to-person variability as possible. Second, although some missing data could be due to testing providers not requiring this information, we recognized that demographic data could be retroactively applied to cases through CI/CT [21]. We included town-level covariates in the regression model to account for potential differences between towns in how they conducted CI/CT. For example, towns with greater median incomes may have larger budgets allocated to LBOH and surveillance epidemiology to enable additional case demographic information ascertainment. Finally, we included number of tests conducted per capita and an indicator variable for weeks of town-level case surges to account for potential infrastructural challenges both in testing and contact tracing during periods of intense case growth when time and resources may be more limited.

### Data Sources

#### Individual-Level COVID-19 Case Data

We obtained individual-level, PCR-confirmed COVID-19 data for all cases through mid-February 2021 in MA through a data-use agreement between the Massachusetts Department of Public Health (MDPH) and the Boston University School of Public Health. The individual-level characteristics of interest from this dataset were race (“American Indian or Alaska Native,” “Asian,” “Black or African American,” “Native Hawaiian or Pacific Islander,” “Other,” “White,” “Unknown,” or left blank), ethnicity (“Hispanic, Latinx, or Spanish”; “Not Hispanic, Latinx, or Spanish”; “Refused”; “Unknown”; or left blank), age (in years), and gender (“Male,” “Female,” “Transgender,” or “Unknown”). We additionally used geolocation in ArcGIS Pro (©Esri, Redlands, CA) to identify institutional-residency cases originating from long-term care facilities (including nursing homes, rest homes, and assisted-living facilities), prisons, and homeless shelters. We created a binary variable of institutional residency and note that the majority of institutional cases included in our model (80.1%) were from long-term care facilities. Although data-collection practices may vary between these types of settings, we chose to keep institutional cases aggregated both for statistical power and to make generalizations about data missingness in congregate residential settings of vulnerable populations. Race and ethnicity for cases were considered missing if the respective field was left blank or marked as “Unknown.” We recoded age as missing for values less than zero or greater than 110 (a reporting error affecting only 0.08% of cases). No algorithmic imputation of missing data was done; all race and ethnicity information recorded for cases was either self-reported by the patient, recorded by the entity conducting the test, or reported during contact-tracing interviews. The case data do not include information on where the testing was done, which lab processed the sample, who provided the race and ethnicity data, or at which point in the process the race and ethnicity data were recorded.

#### Town-Level Data

We obtained the most recently available census-tract-level data from the 5-year (2015–2019) American Community Survey (ACS) [22] and used population weighting to crosswalk the values to the 351 cities and towns of MA. The ACS variables included in the model were median household income, percent population identifying as Black or African American (of any ethnicity), percent population identifying as Hispanic or Latino (of any race, hereafter “Latinx”), and population density (households per unit land area).

We obtained the number of COVID-19 tests performed each week by city/town from the weekly public health reports published by MDPH [23]. We divided the time period into three “phases” to reflect different COVID-19 caseloads, as well as testing and reporting infrastructure over time. The three phases were defined as “Phase 1” (March 1, 2020—June 6, 2020), which covered the initial peak wave of COVID-19 case increases in MA and the state of emergency declaration; “Summer” (June 7, 2020–September 12, 2020), which saw consistently low caseloads during a time period before return to schools and universities; and “Phase 2” (September 13, 2020–February 6, 2021 [the last full week of individual-level data available at the time of analysis]), which included the second wave increase in COVID-19 in MA. Weekly tests by town were not available for most of Phase 1, so we included testing data only for the Summer and Phase 2 models. For each week, we calculated the tests per capita as the number of COVID-19 tests divided by the total population of the town. To assess whether periods of rapid case growth were associated with increased odds of missing race and ethnicity data, we developed an indicator variable for “case surge” for each week in every town. We defined a case as having occurred during a case surge if that week’s percent change in town-level cumulative cases exceeded the 75th percentile of weekly changes in town-level cumulative cases specific to that phase.

### Statistical Analysis

We modeled the likelihood of an individual, non-fatal case having missing race or ethnicity data using mixed-effects logistic regression models, fitting separate models for each phase of the pandemic and for missingness in either the race or ethnicity variable separately. We excluded fatal cases from the statistical analysis (15,500 fatal cases out of 530,854 total cases, or 2.9% of observations) because the process for race/ethnicity ascertainment is different and, as a result, there is extremely little missingness (0.15% missing race and 0.37% missing ethnicity) among fatal case data. The dependent variable was a binary indicator of missingness of either race (in one set of models) or ethnicity (in the other set of models). Fixed effects (described in more detail in “Data Sources” section) occurred on two levels: (1) individual, including age, gender, and institutional residence; and (2) town, including median income, proportion of residents who identify as Black or African American, proportion of residents who identify as Latinx, population density, number of tests per capita during the week the case occurred (included only in the Summer and Phase 2 models, since weekly testing data are not available prior to July 2020), and whether the case occurred during a period of case surge. We used town of case residence as a random effect (intercept only) to account for potentially systematic differences in baseline levels of missingness between towns due to differential capacity for contact tracing (see again the “Conceptual Framework” section). All statistical analyses were done in R (version 4.0.3) [24] using the “glmer” function from the *lme4* package (version 1.1–27.1) [25].

## Results

### Distributions of Missing Race and Ethnicity Data

**Fig. 1:**
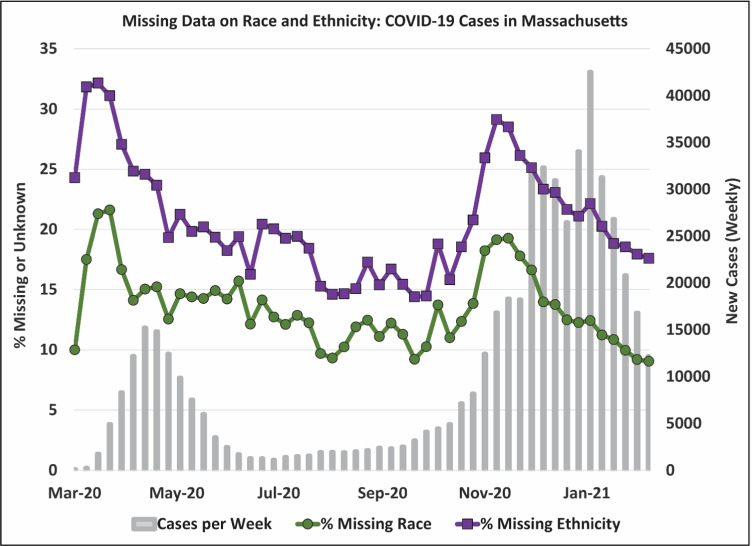
Percent of weekly COVID-19 cases in Massachusetts with unknown or missing race or Latinx ethnicity through early February 2021. The number of weekly new cases since pandemic onset (bars) and percent of cases with missing or unknown race (lower line with circles) or Latinx ethnicity (upper line with squares). Source: Authors’ analysis of COVID-19 surveillance data from the Massachusetts Virtual Epidemiologic Network (MAVEN), maintained by the Massachusetts Department of Public Health

Missing race and Latinx ethnicity data during the COVID-19 pandemic in MA varied over time, with percent missingness following similar patterns as weekly new cases (Fig. 1). The highest percent missingness occurred very early in the pandemic (March 2020: 21.6% for race and 32.2% for ethnicity), though rates approached this maximum again during the period of the highest rate of case growth in the second wave of cases in November 2020 (19.3% for race and 29.1% for ethnicity). The Summer months saw relatively stable lower levels of missing data and similarly low caseloads. Overall, missingness in race and Latinx ethnicity data were highly correlated, with the ethnicity data consistently having greater rates of missingness than the race data across all weeks.

**Fig. 2:**
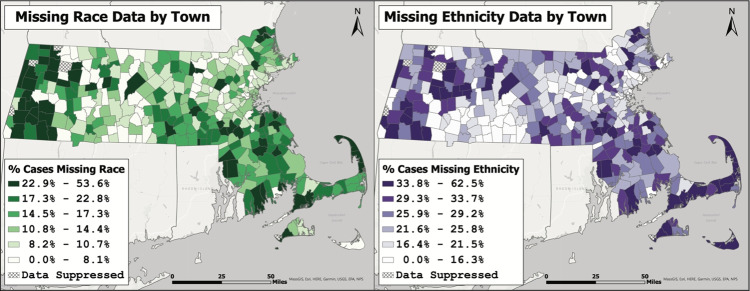
Percent of COVID-19 cases in Massachusetts with unknown or missing race or Latinx ethnicity by town. The percent of lab-confirmed COVID-19 cases in Massachusetts as of early February 2021 with missing or unknown race (left map in green) and Latinx ethnicity (right map in purple) by town. Source: Authors’ analysis of COVID-19 surveillance data from the Massachusetts Virtual Epidemiologic Network (MAVEN), maintained by the Massachusetts Department of Public Health

In addition to changes over time, there were also substantial differences in missingness among towns (Fig. 2). South-eastern and western regions of MA had amongst the highest percentages of missing race and Latinx ethnicity data, while south-central and eastern MA (including Boston and surrounding towns) had amongst the lowest. Relative spatial patterns of missingness were similar between race and Latinx ethnicity.

### Individual- and Town-Level Determinants of Missingness

**Fig. 3:**
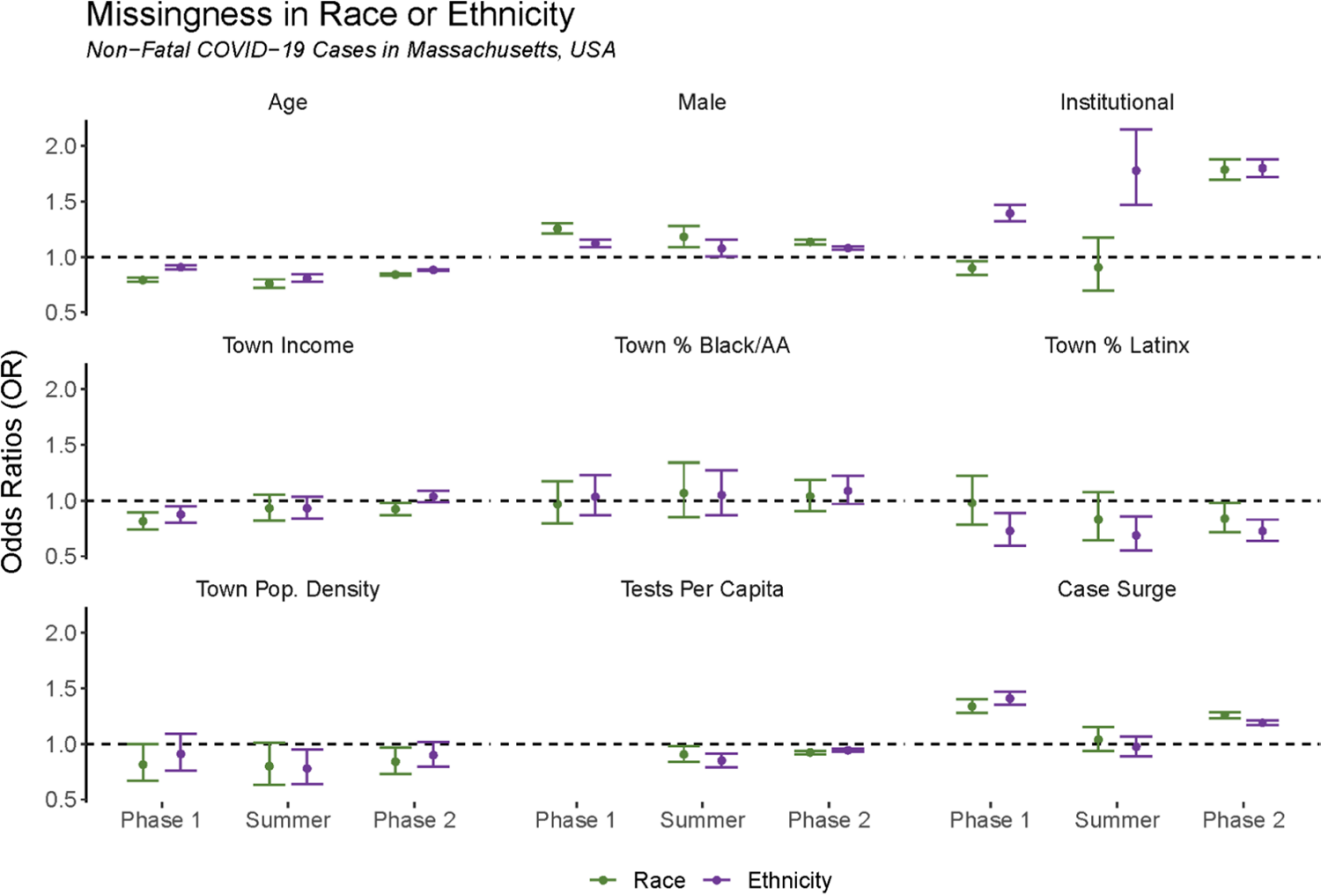
Associations between individual- and town-level characteristics and missing race and ethnicity data on non-fatal COVID-19 cases in Massachusetts, March 2020 – February 2021. Exponentiated coefficients (odds ratios) from mixed-effects regressions modeling the probability that a non-fatal COVID-19 case in Massachusetts would have missing/unknown race (green points, on the left of each pair) or ethnicity (purple points, on the right of each pair) by phase of pandemic. “Phase 1” is from March 1, 2020–June 6, 2020, “Summer” is from June 7, 2020–September 12, 2020, and “Phase 2” is from September 13, 2020–February 6, 2021. Error bars represent 95% confidence intervals. Weekly testing data are not available for Phase 1 Abbreviations: *Pop* population and *AA* African American. Source: Authors’ analysis of COVID-19 surveillance data from the Massachusetts Virtual Epidemiologic Network (MAVEN), maintained by the Massachusetts Department of Public Health

Our mixed-effects models identified several individual- and town-level characteristics that were significantly associated with the odds of a COVID-19 case having missing information on race or ethnicity (Fig. 3 and Table 1). Age was significantly associated with *lower* odds of missingness in race and ethnicity across all time periods (i.e., older cases were less likely to have missing race and ethnicity characteristics; odds ratios (ORs, expressed here as estimate [95% confidence interval]) ranged from 0.76 [0.72, 0.80] for race during the Summer to 0.91 [0.89, 0.92] for ethnicity in Phase 1), while male gender was significantly associated with *greater* odds across all time periods (i.e., male cases were more likely to have missingness; ORs ranged from 1.08 [1.06, 1.10] for ethnicity in Phase 2 to 1.25 [1.21, 1.30] for race in Phase 1). Institutional residency status was very strongly associated with missing *ethnicity* data across all time periods (ORs ranged from 1.39 [1.32, 1.47] in Phase 1 to 1.80 [1.72, 1.88] in Phase 2); however, although institutional residency status was similarly associated with missing race during Phase 2, it was statistically significantly *negatively* associated with missing race data during Phase 1 (OR = 0.90 [0.84, 0.96]) and not statistically significantly associated during the Summer (OR = 0.91 [0.70, 1.18]).

**Table 1:** Associations between individual- and town-level characteristics and missing race and ethnicity data on non-fatal COVID-19 cases in Massachusetts, March 2020 – February 2021. Exponentiated coefficients (odds ratios) from mixed-effects regressions modeling the probability that a non-fatal COVID-19 case in Massachusetts would have missing/unknown race or ethnicity by phase of pandemic. “Phase 1” is from March 1, 2020–June 6, 2020, “Summer” is from June 7, 2020–September 12, 2020, and “Phase 2” is from September 13, 2020–February 6, 2021. Weekly testing data are not available for Phase 1. Bold font indicates that the OR is statistically significant at the 5% level prior to rounding to two decimal places. See also Fig. 3, which displays these data graphically

		Exponentiated coefficients (odds ratios) and 95% confidence intervals					
		Phase 1		Summer		Phase 2	
Covariates		Race	Ethnicity	Race	Ethnicity	Race	Ethnicity
Individual-level							
	Age	**0.79 (0.77, 0.81)**	**0.91 (0.89, 0.92)**	**0.76 (0.72, 0.80)**	**0.81 (0.78, 0.84)**	**0.84 (0.83, 0.85)**	**0.88 (0.88, 0.89)**
	Male	**1.25 (1.21, 1.30)**	**1.12 (1.09, 1.16)**	**1.18 (1.09, 1.28)**	**1.08 (1.00, 1.15)**	**1.14 (1.12, 1.16)**	**1.08 (1.06, 1.10)**
	Institutional residency	**0.90 (0.84, 0.96)**	**1.39 (1.32, 1.47)**	0.91 (0.70, 1.18)	**1.78 (1.47, 2.15)**	**1.78 (1.69, 1.88)**	**1.80 (1.72, 1.88)**
Town-level							
	Median income	**0.81 (0.74, 0.90)**	**0.87 (0.80, 0.95)**	0.93 (0.82, 1.05)	0.93 (0.84, 1.03)	**0.92 (0.87, 0.98)**	1.04 (0.99, 1.09)
	% Black or African American	0.97 (0.80, 1.17)	1.03 (0.87, 1.23)	1.07 (0.85, 1.34)	1.05 (0.87, 1.27)	1.04 (0.91, 1.19)	1.09 (0.97, 1.22)
	% Latinx	0.98 (0.78, 1.22)	**0.73 (0.59, 0.89)**	0.83 (0.64, 1.08)	**0.69 (0.55, 0.86)**	**0.84 (0.72, 0.98)**	**0.73 (0.64, 0.83)**
	Population density	**0.81 (0.67, 1.00)**	0.91 (0.76, 1.09)	0.80 (0.63, 1.01)	**0.78 (0.64, 0.95)**	**0.84 (0.73, 0.97)**	0.90 (0.80, 1.01)
	Tests per capita	-	-	**0.90 (0.84, 0.98)**	**0.85 (0.79, 0.91)**	**0.92 (0.91, 0.94)**	**0.94 (0.93, 0.95)**
	Case surge	**1.34 (1.28, 1.40)**	**1.41 (1.35, 1.47)**	1.04 (0.93, 1.15)	0.97 (0.89, 1.07)	**1.26 (1.23, 1.29)**	**1.19 (1.17, 1.21)**

Among the town-level characteristics, the variable with the greatest magnitude of association was the indicator for “case surge,” which was significantly positively associated with missing race and ethnicity in both Phase 1 and Phase 2 (ORs ranged from 1.19 [1.17, 1.21] for ethnicity in Phase 2 to 1.41 [1.35, 1.47] for ethnicity in Phase 1); we observed no association during the Summer, consistent with the lack of rapid case growth during this time (see again Fig. 1). Number of tests administered per capita had a negative association with missingness during the Summer (ORs = 0.90 [0.84, 0.98] for race and 0.85 [0.79, 0.91] for ethnicity) and in Phase 2 (ORs = 0.92 [0.91, 0.94] for race and 0.94 [0.93, 0.95] for ethnicity). Median income was negatively associated with missingness during Phase 1 (ORs = 0.81 [0.74, 0.90] for race and 0.87 [0.80, 0.95] for ethnicity), but it had null or marginal effects for the other periods. Although town population proportions of Black or African American residents showed no association with missingness during any period, towns with greater shares of Latinx residents were significantly less likely to have missing ethnicity data during all periods (ORs ranged from 0.69 [0.55, 0.86] during the Summer to 0.73 [0.64, 0.83] during Phase 2), and significantly less likely to have missing race data during Phase 2 (OR = 0.84 [0.72, 0.98]), but no statistically significant effect for the two earlier periods.

## Discussion

Substantial amounts of missing data on race and Latinx ethnicity among COVID-19 cases in MA occurred during the first year of the pandemic, with strong variability over time and among towns. This missingness occurred despite calls from the public for greater tracking of racial and ethnic disparities in COVID-19 [26], as well as state and federal requirements to report demographic data [27, 28]. This extent of missingness suggests that complete demographic data collection for COVID-19 is complicated and underscores existing challenges for surveillance epidemiology that advances health equity. In this paper, we found multiple characteristics across spatial scales associated with missing data, potentially indicative of a variety of individual, infrastructural, and situational causes of missingness.

One source of missing race and ethnicity data in COVID-19 cases is a reluctance among some individuals to provide the information, which could be due to privacy concerns, prior experiences of racism in medical treatment, concerns about immigration status, lack of representativeness of their specific identity in the choices provided on the form, or other sources of hesitancy. Baker et al. found that 28% of surveyed patients were uncomfortable providing their race and ethnicity to healthcare providers [29], and Kandula et al. found that such reluctance is greater among people who have experienced discrimination [30]. Patients are generally more comfortable providing their race and ethnicity when the healthcare provider explains that it is being collected to monitor quality of care versus other rationales, including that it is mandatory to collect [29, 31]. During growth phases of the pandemic when many people may have been tested at large-scale or drive-through testing sites outside of a healthcare setting, it was more challenging to have the capacity to discuss the rationale for race and ethnicity data collection. Since many COVID-19 testing sites in MA and elsewhere—including at retail pharmacies and mass-testing sites—collect race and ethnicity data via a form without a stated rationale, a potential mechanism to increase response rates could be to add a brief explanation that the demographic data are collected for the purpose of identifying and addressing health inequities and have no impact on the availability or quality of care. Similar clear-language statements underscoring HIPAA legal protections may assuage some privacy concerns and encourage greater response rates. Finally, strategies to promote more granularity of racial and ethnic categories for self-identification, such as allowing space for free-form responses [32], may also increase response rates. This may be particularly applicable to Latinx individuals, who may be less likely to specify a racial identification in healthcare settings [33]. Moreover, a template for this kind of increased granularity already exists: in 2011, the US Department of Health and Human Services (HHS) issued new internal standards of data collection for HHS-sponsored surveys that expanded the number of identities listed within “Asian,” “Native Hawaiian or Pacific Islander,” and “Hispanic or Latino” identities included in the federal data standards set by the Office of Management and Budget (OMB) [4]. Wide adoption of these existing but underutilized HHS standards for local- and state-level public health surveillance could potentially improve response rates on patient demographic questionnaires and advance goals of health equity.

We found that age and gender were both significant predictors of missing race and ethnicity data during the first year of the pandemic in MA. We observed a negative association for age and a positive association for male gender, which could indicate a greater hesitancy to provide this information among younger and/or male individuals. However, these findings are only somewhat consistent with the limited available research on age- and gender-stratified attitudes toward providing demographic information in healthcare settings: while one study found that female patients may be more comfortable providing their preferred language than male patients [34], the evidence suggests more reluctance to provide ethnicity among older patients [34, 35], counter to our findings. An alternative explanation is that contact tracing is more difficult to successfully complete among younger patients, due to difficulty in reaching them via telephone or a reluctance to participate. It is also possible that more-severe cases, which occur more frequently among older patients, could result in less missingness due to there being more points of data entry: a patient who first gets tested at a retail pharmacy and then is tested again when seeking medical care for worsening symptoms will be asked twice to provide demographic data, compared to just once for someone who tests positive but does not seek medical attention.

Among the individual-level predictors, we found a particularly strong positive association between missing ethnicity data and institutional residency across all time periods, and a similar association with missing race during Phase 2. In contrast to testing that occurs outside of institutional residences—most commonly conducted at retail pharmacies or mass-testing sites with self-completed questionnaires—institutional residents receiving a COVID-19 test may have it administered by a healthcare provider who already routinely works with the resident. Although this could provide an opportunity to explain the rationale for gathering race/ethnicity data, some studies have shown that many healthcare providers are uncomfortable asking patients for their race or ethnicity [36]. If this is the case, then policymakers and public health agencies may need to better communicate the rationale for data-collection mandates and work with healthcare employers on integrating this into professional training, onboarding procedures, and continuing education requirements.

But individual factors are unlikely to be the sole drivers of missing race and ethnicity data in COVID-19 cases, as reflected in the significant associations we found with several town-level variables. Of particular importance is the role of case burdens. Given our finding that weeks of case surges were associated with significantly greater odds of missingness, we hypothesize that race and ethnicity data collection faltered when healthcare and public health systems were strained. The statewide data show that missingness was greatest during the early periods of case outbreaks and peaked *prior to* the weekly maxima of caseloads, suggesting challenges specific to ramping up testing capacity. This could include time constraints from understaffing, the involvement of new staff or settings, or a variety of other factors consistent with overstressed healthcare and public health infrastructure. In the context of a public health state of emergency, activation of the National Guard [37] and other emergency-use changes can present additional challenges to data collection. While we did not specifically assess this here, we posit that development of robust, consistent, and easy-to-use reporting systems (with associated targeted training) may help future data collection efforts in similar contexts.

We found that other town-level characteristics were also predictors of missingness, which we hypothesize could be due to differences in contact tracing capacities between local boards of health. Our finding that towns with greater proportions of residents who identify as Latinx have lower rates of missing ethnicity data could suggest greater cultural competencies on the part of the LBOH employees during contact tracing. For example, they may be more attuned to the health disparities affecting the Latinx communities in their town (therefore recognizing the importance of collecting demographic data to document it), or the LBOH staff may have more multilingual capabilities to enable communicating in the patient’s preferred language.

While we could not formally assess data collection technologies in our study, the persistent race/ethnicity missingness indicates that there are likely both technological barriers and technological solutions that could reduce missingness. Complex reporting systems, varying collection forms, systems that do not allow for self-reporting of identity, and inconsistencies in data management infrastructure all pose impediments to the collection of race and ethnicity data, especially when more testing sites and laboratories are rapidly engaged, such as during an upswing in COVID-19 cases. For example, some diagnostic laboratories reporting COVID-19 test results to MDPH do not capture race and ethnicity at all in their systems, leading to missingness in the final dataset reported to the state. One potential way to address this would be to enforce a mandate on the collection of race and ethnicity data from specimen laboratories; this would build upon a 2007 regulation that required acute-care hospitals in MA to collect self-reported race and ethnicity data [38], which nearly doubled the number of hospitals collecting these data in just two years [39]. Continued efforts to improve interoperability of electronic health records [40], including the wider adoption of electronic case reporting (eCR) [41], may improve efficiency of data collection. These technological upgrades to health data collection and reporting could be made possible in the near future with investments from the American Rescue Act of 2021 (sometimes informally referred to as the COVID-19 Stimulus Package), which allocated billions of federal dollars to states for a variety of economic recovery and infrastructural improvement purposes in response to the COVID-19 pandemic [42]. When allocating funds received by Massachusetts from this stimulus, the state legislature stipulated that “not less than $98,850,000 shall be expended to establish standardized and unified data systems to increase capacity to collect, analyze and share data to protect the public’s health and evaluate system performance” [43].

Despite all the challenges discussed here, we know that it is possible to get nearly complete demographic data on cases of infectious diseases based on experiences from other epidemics. An example is HIV surveillance, which maintains high levels of completeness of race and ethnicity data, related in part to long-term linkages with the healthcare system, federal funding for follow-up, required reporting of race and ethnicity in order to be included in official case counts [44], and allocation of federal funds based on case counts [45]. While these structures are not applied for surveillance of many infectious diseases, a generalizable lesson would be that increased support for local health departments, coupled with concrete incentive structures, may yield more-complete demographic data collection. An important caveat suggested by this study—that it remains difficult to implement systems during periods of rapid case growth—may warrant additional interventions.

## Data Availability

The COVID-19 outcome data were made available to the authors by the Massachusetts Department of Public Health under a data-use agreement and cannot be shared by the authors. The community-level covariate data from the American Community Survey and other public sources are available from the corresponding author upon request.

## Abbreviations

COVID-19: Coronavirus disease 2019
MA: Massachusetts
MDPH: Massachusetts Department of Public Health
MAVEN: Massachusetts Virtual Epidemiologic Network
eCR: Electronic case reporting

## Code Availability

The code used in this analysis is available from the corresponding author upon request.
